# Involvement of spinal α_2_‐adrenoceptors in prolonged modulation of hind limb withdrawal reflexes following acute noxious stimulation in the anaesthetized rabbit

**DOI:** 10.1111/ejn.13185

**Published:** 2016-02-28

**Authors:** John Harris

**Affiliations:** ^1^ School of BiosciencesUniversity of NottinghamSutton Bonington CampusLoughboroughLE12 5RDUK

**Keywords:** descending inhibition, DNIC, mustard oil, RX 821002, spinal cord

## Abstract

The role of spinal **α**_2_‐adrenoceptors in mediating long‐lasting modulation of hind limb withdrawal reflexes following acute noxious chemical stimulation of distant heterotopic and local homotopic locations has been investigated in pentobarbitone‐anaesthetized rabbits. Reflexes evoked in the ankle extensor muscle medial gastrocnemius (MG) by electrical stimulation of the ipsilateral heel, and reflexes elicited in the ankle flexor tibialis anterior and the knee flexor semitendinosus by stimulation at the base of the ipsilateral toes, could be inhibited for over 1 h after mustard oil (20%) was applied to either the snout or into the contralateral MG. The heel–MG response was also inhibited after applying mustard oil across the plantar metatarsophalangeal joints of the ipsilateral foot, whereas this homotopic stimulus facilitated both flexor responses. Mustard oil also caused a significant pressor effect when applied to any of the three test sites. The selective α_2_‐adrenoceptor antagonist, RX 821002 (100–300 μg, intrathecally), had no effect on reflexes *per se*, but did cause a decrease in mean arterial blood pressure. In the presence of the α_2_‐blocker, inhibitory and facilitatory effects of mustard oil on reflexes were completely abolished. These data imply that long‐lasting inhibition of spinal reflexes following acute noxious stimulation of distant locations involves activation of supraspinal noradrenergic pathways, the effects of which are dependent on an intact α_2_‐adrenoceptor system at the spinal level. These pathways and receptors also appear to be involved in facilitation (sensitization) as well as inhibition of reflexes following a noxious stimulus applied to the same limb.

## Introduction

In pentobarbitone‐anaesthetized or decerebrated rabbits, withdrawal reflexes of the hind limb are profoundly inhibited by noxious stimulation of certain off‐limb (heterotopic) sites, a finding comparable to counter‐stimulation phenomena such as diffuse noxious inhibitory controls (DNIC; Le Bars *et al*., 1979a,b; Schouenborg & Dickenson, 1985; Villanueva & Le Bars, 1995; Le Bars & Willer, 2002), which more recently has been termed ‘conditioned pain modulation’ (CPM) in humans (Yarnitsky *et al*., 2010). Thus, reflex responses in the ankle extensor medial gastrocnemius (MG) evoked by electrical stimulation of heel afferents, and responses in the ankle flexor tibialis anterior (TA) and the knee flexor semitendinosus (ST) to stimulation of the toes, could be depressed for over 1 h after the chemical irritant mustard oil was applied either to the snout or into the MG muscle of the contralateral hind limb (Harris & Clarke, 2003). In the same studies, administration of mustard oil to off‐limb sites in decerebrated, spinalized animals had no modulatory effect on reflexes, indicating that activation of one or more descending pathways is necessary for this inhibition to occur.

Anatomical tracing studies in the rabbit and other species have shown that a major portion of the bulbospinal innervation of the spinal cord is by fibres containing the monoamines noradrenaline or 5‐hydroxytryptamine (5‐HT; Blessing *et al*., 1978, 1981; Howe *et al*., 1983), descending pathways that are well established in mediating inhibitory (and facilitatory) modulation of spinal nociceptive activity (Millan, 2002; Ossipov *et al*., 2010), making either of these neurotransmitters strong candidates for mediating the spinal effects of mustard oil applied to off‐limb sites. In this respect, studies in the rat using single dorsal horn neurons or pain behaviour have indicated the involvement of descending 5‐HT‐ergic pathways in DNIC (Dickenson *et al*., 1981; Chitour *et al*., 1982; Kraus *et al*., 1982), whilst the possible contribution of noradrenergic pathways has also been suggested by studying dorsal horn neurons or tail flick responses (Gjerstad *et al*., 2000; Wen *et al*., 2010). The nature of the descending pathways involved in heterotopic inhibition of specific limb flexor and extensor withdrawal reflexes has received little attention, however, given the potential for reflex responses to be differentially modulated by these pathways (Harris & Clarke, 2003). Because it has previously been shown that inhibition of the heel–MG reflex by high‐intensity electrical stimulation of nerves in the forelimbs and hind limbs is reduced by intravenous administration of the α_2_‐adrenoceptor antagonist idazoxan (Taylor *et al*., 1991), the present studies have therefore focused on the spinal role of noradrenergic pathways in mediating the effects of remotely applied mustard oil by intrathecally applying a derivative of idazoxan, RX 821002 [2‐methoxy‐idazoxan; 2‐(2,3‐dihydro‐2‐methoxy‐1,4‐benzodioxin‐2‐yl)‐4,5‐dihydro‐1*H*‐imidazole hydrochloride]. Like its parent compound, RX 821002 is a selective α_2_‐adrenoceptor antagonist (Stillings *et al*., 1985; Welbourn *et al*., 1986) but, in contrast to idazoxan, it does not possess any appreciable affinity for non‐adrenergic imidazoline receptors (Hudson *et al*., 1992; Clarke & Harris, 2002).

As well as off‐limb sites, long‐lasting modulation of hind limb reflexes by mustard oil can also be induced from the same limb, but the effect observed is highly dependent upon the part of the limb stimulated and the reflex studied (Harris & Clarke, 2003). For instance, mustard oil application to the plantar metatarsophalangeal (MT) joints leads to inhibition of heel–MG reflex responses but, contrary to this, flexor responses are subject to prolonged facilitation. This facilitation of flexor responses is restricted to just the plantar surface of the foot in intact animals, whereas facilitation can be produced from all over the hind limb in spinalized animals, indicating a role for tonic inhibitory pathways in spatially restricting reflex sensitization so that only functionally protective withdrawal responses are enhanced (Harris & Clarke, 2003). There is a wealth of evidence to indicate that descending noradrenergic pathways inhibit nociceptive transmission in the spinal cord via α_2_‐adrenoceptors (Millan, 2002; Pertovaara, 2006), and that a reduction in this inhibition contributes to hypersensitivity seen in the injured limb in models of acute inflammation (Green *et al*., 1998; Omote *et al*., 1998; Mansikka *et al*., 2004) and nerve injury (Xu *et al*., 1999; Wei & Pertovaara, 2006; Rahman *et al*., 2008; De Felice *et al*., 2011; Hughes *et al*., 2013); however, again little is known about how these pathways may be altered to influence cutaneo‐muscular reflexes in individual flexor and extensor muscles. Therefore, the effects of spinal administration of RX 821002 on mustard oil‐induced modulation of hind limb reflexes following its application to the plantar MT joints (i.e. a homotopic site) have also been investigated in the same preparation in the present studies.

## Materials and methods

### General preparation

Experiments were performed on 19 rabbits of mixed strain and either sex, weighing between 1.8 and 3.5 kg, in accordance with the EC Directive 86/609/EEC for animal experiments, UK Animals (Scientific Procedures) Act of 1986, and following approval by the University of Nottingham ethical committee. Animals were sedated with 50 mg i.m. ketamine sulphate (Fort Dodge Animal Health, Southampton, UK) before pentobarbitone sodium (mean dose 45 mg/kg over 20 min, range 35–55 mg/kg) was administered via a marginal ear vein to induce anaesthesia. The pentobarbitone sodium solution (30 mg/mL in Ringer's) was diluted from a stock solution (60 mg/mL) prepared by dissolving powdered sodium pentobarbital (6% w/v) in 20% v/v propylene glycol, 10.4% v/v ethanol (96%) and distilled water (all constituents from Sigma‐Aldrich, UK). All areas subject to surgery were pre‐treated with 2% lignocaine solution s.c. or i.m. (about 100 μL per injection; Lignavet, C‐Vet Veterinary Products, Leyland, UK), and following surgery lignocaine ointment (5%; Biorex Laboratories, Enfield, UK) was applied to any cut muscle and skin surfaces. The trachea was cannulated, then the left carotid artery and left jugular vein were also cannulated to allow the measurement of arterial blood pressure and administration of intravenous drugs, respectively. Anaesthesia was maintained using a continuous i.v. infusion (at a mean rate of 17 mg/kg/h, range 13–24 mg/kg/h) of pentobarbitone sodium (diluted from stock solution to 6 mg/mL using 100 mm d‐glucose, 100 mm NaHCO_3_ solution; Sigma‐Aldrich, UK). From this point, animals were artificially ventilated on room air supplemented with oxygen using a Harvard small animal ventilator with the stroke volume set to maintain end tidal CO_2_ between 3.5 and 4.5%. To allow direct spinal (intrathecal) application of drugs to the lumbosacral spinal cord, a fine polyethylene cannula (o.d. = 0.63 mm; Portex, Smiths Medical, Ashford, UK) was threaded caudally beneath the dura mater via a laminectomy at the thoracolumbar junction. No other invasive surgery was performed. The core temperature of the animal was measured using a rectal probe and maintained at 38 ± 0.5 °C by a thermostatically controlled heating blanket. An electrocardiogram was recorded using an intra‐oesophageal probe, and this triggered an instantaneous rate meter to provide a measurement of heart rate. The output from this rate meter, as well as the amplified signal from a pressure transducer connected to the cannula in the carotid artery, were continuously recorded on a computer linked to a Cambridge Electronic Design (CED, Cambridge, UK) micro1401 interface and running Spike2 for Windows v.3.

### Stimulation and recording of reflexes

To evoke reflexes, paired stainless‐steel needle electrodes were inserted into the skin of the plantar surface of the left foot at the heel and at the base of the toes. Blocks of eight constant‐current stimuli at 1 Hz and of 1 ms duration were then applied from AMPI Isoflex stimulators alternately to the heel and toe electrodes at 2 min intervals. In most cases, stimulus strength was set at 1–5 times the threshold value for evoking reflex responses; however, in two experiments (in one case for the heel–MG response and the other for toes–ST or toes–TA responses) it was not possible to evoke measurable reflexes using eight single shocks at a stimulus strength up to 10 mA. Therefore, in these two instances, eight triple shocks (at 250 Hz) were given every second at strengths of 4.1 mA and 10 mA to evoke heel–MG responses and toes–ST or toes–TA responses, respectively. The stimulus strength in each experiment was based on obtaining reflex responses that had the capacity to measurably increase or decrease in size following the conditioning stimuli (Harris & Clarke, 2003; Harris *et al*., 2004). To this end, a small increase in stimulation intensity was occasionally necessary during a control period between conditioning stimuli (i.e. after at least 1 h of post‐conditioning responses had been measured and reflexes had stabilized; see below).

**Figure 1:**
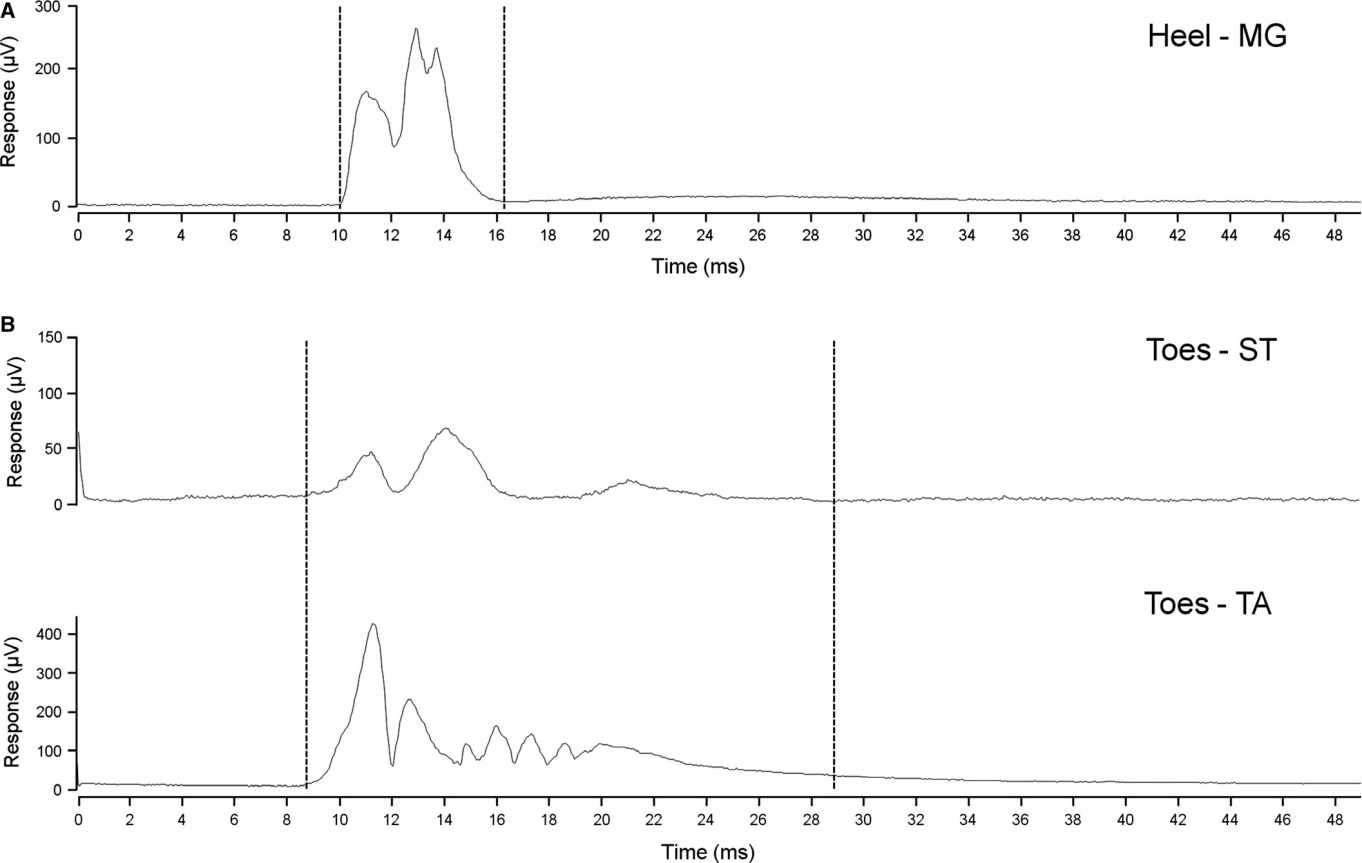
Example electromyogram recordings and analysis time windows of reflex responses in (A) medial gastrocnemius (MG) and (B) semitendinosus (ST)/tibialis anterior (TA) to electrical stimulation of the heel and toes, respectively. Each record is the average of eight sweeps and the stimulus was applied at the beginning of each sweep. Placement of cursors for measurement of the voltage/time integral (area) of the response is indicated by the dashed lines.

Reflex responses to heel stimulation were recorded as compound electromyogram signals from the ipsilateral MG extensor muscle using paired, percutaneous, varnish‐insulated copper wire electrodes. Reflexes to toe stimulation were recorded in the same way from ipsilateral TA and ST flexor muscles. Signals were amplified (2000–5000 times) and filtered (between 1 Hz and 6 kHz), then digitized using a CED 1401 interface before the full‐wave rectified responses to each block of eight stimuli were averaged and integrated by computer using Signal v.2.12. As the duration of reflexes can vary slightly between experiments, the analysis window was determined for an individual experiment rather than fixed for all experiments. Measurement (voltage/time area) of short‐latency heel–MG responses was therefore performed between 9–12 ms (latency of response) and 14–27 ms (return of response to baseline, i.e. duration), whilst the corresponding analysis window for toes–flexor responses was from 9–13 ms to 23–42 ms (Fig. 1).

### Experimental protocol

Control reflex responses to heel and toe stimulation were recorded for at least 30 min before the first noxious conditioning stimulus was applied. This consisted of 100 μL 20% mustard oil (allylisothiocyanate; Sigma‐Aldrich, UK) in paraffin oil, which was applied to one of three sites: onto the skin of the plantar surface of the ipsilateral foot across the MT joints; onto the skin of the snout; or it was injected into the contralateral MG muscle. Heel–MG and toes–flexor reflexes were then measured for at least 1 h before mustard oil was applied to one of the two previously unstimulated areas and the recording period repeated. An identical procedure was followed after the final site received the noxious conditioning stimulus. The order in which mustard oil was applied to the three sites was randomized for each experiment. Subsequent to the first round of mustard oil stimuli, 10 animals (hereafter referred to as the RX 821002 treatment group) were given the selective α_2_‐adrenoceptor antagonist, RX 821002 (Tocris, Bristol, UK) dissolved in Ringer's solution, intrathecally at a dose of 200 μg (seven animals) or 300 μg (three animals) from stock solutions of 2 mg/mL and 3 mg/mL, respectively (flushed in with 50 μL Ringer's), whilst nine animals received no drug treatment (hereafter referred to as the control (no drug) group). Previous studies have shown that intrathecal Ringer's has no effect on reflexes *per se* (Harris & Clarke, 1992; Clarke *et al*., 2001a). The relatively high initial intrathecal dose of RX 821002 was chosen to ensure an effective local concentration of antagonist was achieved at α_2_‐adrenoceptors in the spinal cord following permeation into the grey matter. Reflexes were then recorded for between 24 and 44 min until responses had become stable, then a second round of mustard oil stimuli and reflex recording was initiated. For each of the three stimulus sites, the second application of mustard oil was given adjacent to, but not directly on top of, the site of the first mustard oil treatment. Very few data have been published on the pharmacokinetics of RX 821002 (Clarke & Harris, 2002), and no definitive measurements have been made of duration of action, but previous use has shown that it has effects that last longer than 1 h in the rabbit. Therefore, in the group of animals receiving RX 821002, booster doses at half the initial concentration (i.e. 100 or 150 μg, intrathecally) were administered before mustard oil was applied to the second and third sites. Experiments were terminated by giving an overdose of anaesthetic followed by 2 mL of saturated KCl solution.

### Data handling and statistical analysis

The effect of mustard oil on reflex responses was determined by expressing post‐mustard oil readings as a percentage of the mean of the three responses recorded immediately prior to the conditioning stimulus. For each conditioning stimulus, the time taken for reflex responses to recover (to within 2 standard deviations of the mean pre‐mustard oil control values for two consecutive readings) was also determined, with cut‐off values of 61 min for the heel–MG reflex and 63 min for the flexor reflexes. Because reflex responses are generally not normally distributed, values within treatment groups are expressed as medians with inter‐quartile ranges (IQRs), and the data have been analysed with non‐parametric statistical tests using GraphPad Prism v.5.02 (Graph Pad, San Diego, CA, USA). Blood pressure and heart rate data were obtained from the Spike2 records by measuring average values over 1‐min time bins. Because arterial blood pressure and heart rate data were normally distributed, standard parametric tests were used to assess statistical significance. A significance level of 0.05 was assumed throughout.

## Results

### Stimulus intensities for evoking reflexes

The median threshold stimulus intensities required in control (no drug) and RX 821002 treatment groups to evoke reflex responses in MG from the heel, and in ST and TA from the toes, were not significantly different (Mann–Whitney tests, *P *> 0.05), so these data have been pooled. Therefore, for all animals (i.e. *n* = 19), heel–MG responses were evoked at a median threshold stimulus intensity of 3 mA (range 0.6–10 mA), and the corresponding value for toes–flexor responses was 1.5 mA (range 0.5–10 mA). Note that in both cases, the upper range limit of 10 mA was the result of assigning this threshold value to experiments where reflexes could not be evoked using single shocks (see Materials and methods). Statistical comparison of these data confirmed previous findings in pentobarbitone‐anaesthetized rabbits, that the threshold for evoking reflexes in MG from the heel was significantly greater (Wilcoxon test, *P *< 0.05) than that for evoking responses in TA and ST from the toes, and may be due to a greater reduction in descending inhibitory control of flexor compared with extensor reflexes in this preparation (Harris & Clarke, 2003).

### The effects of intrathecal administration of the α_2_‐adrenoceptor antagonist RX 821002 per se

**Figure 2:**
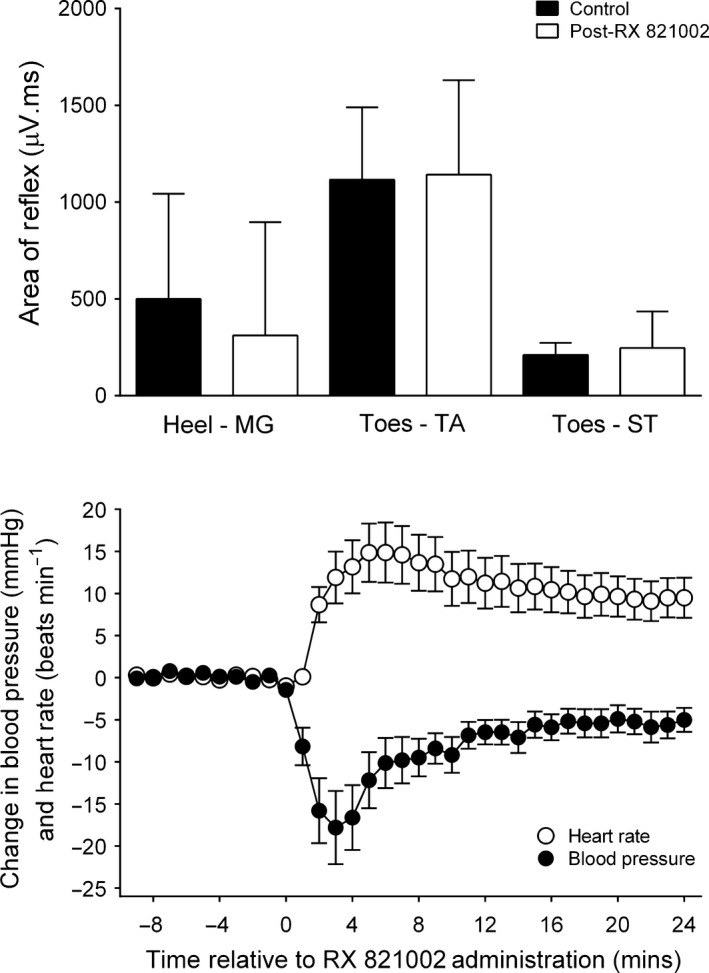
Effect of the selective α_2_‐adrenoceptor antagonist RX 821002 on reflex responses (top panel) and cardiovascular parameters (bottom panel) (*n* = 10). For reflexes, column height represents the median response and error bars are 1st quartile values. For cardiovascular data, points are means and error bars are SEM. RX 821002 was given intrathecally (200–300 μg) at time 0.

There were no obvious differences in the effects of the 200 μg and 300 μg intrathecal doses of RX 821002, so these data have been pooled (i.e. *n* = 10). RX 821002 *per se* had no consistent or significant effect on any of the reflexes studied [Friedman's anova,* F* = 4.714 (MG), 4.671 (TA), 6.729 (ST); *P *> 0.3 in each case]; however, it did have a significant effect on cardiovascular parameters [repeated‐measures anova,* F*_20,180_ = 6.5 (BP), *F*_20,180_ = 11 (HR); *P *< 0.0001 for both; Fig. 2]. Three minutes after RX 821002 administration, mean arterial blood pressure decreased by an average of 18 ± 4 mmHg from a pre‐drug level of 90 ± 4 mmHg, whereas heart rate subsequently increased by 12 ± 3 beats/min from a pre‐RX 821002 level of 300 ± 9 bpm. Blood pressure and heart rate returned to within control levels after a mean of 17 ± 3 min and 22 ± 2 min, respectively.

### The effect on reflex responses of mustard oil injected into the contralateral MG muscle

**Figure 3:**
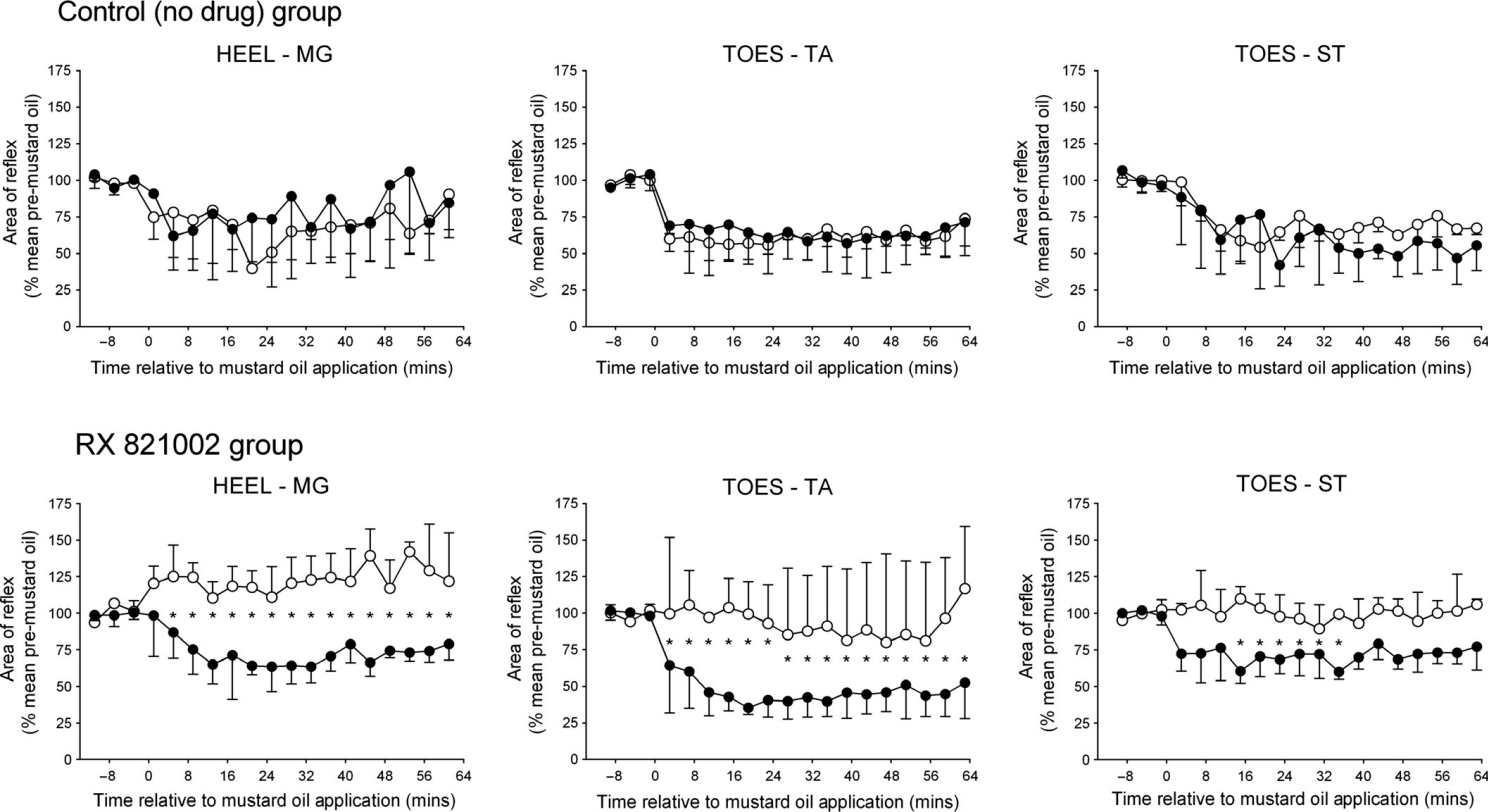
Effect of repeated mustard oil application to the contralateral medial gastrocnemius (MG) muscle (• first injection; ∘ second injection) on heel–MG, toes–tibialis anterior (TA) and toes–semitendinosus (ST) reflexes in the absence (top panels) or presence (bottom panels) of RX 821002 (100–300 μg, intrathecally) prior to the second injection. Each point on the graphs represents the median of nine (no drug group) or 10 (RX 821002 group) experiments, and the vertical lines are either 1st or 3rd quartiles. Mustard oil was applied at 0 min. *Indicates a significant difference in the effect of mustard oil at equivalent time points before and after intrathecal RX 821002 (Wilcoxon tests, *P *< 0.05).

When injected for the first time, mustard oil into the contralateral MG significantly inhibited all three reflexes in both treatment groups [Friedman's anova, RX 821002 treatment group: *F* = 25.33 (MG), 41.84 (TA), 26.40 (ST); control (no drug) group: *F* = 16.00 (MG), 17.72 (TA), 16.09 (ST); *P *< 0.05 in all cases]. Although a decrease in responses was apparent immediately after injection of the mustard oil, in all cases the full effect of this noxious stimulus did not develop until at least 10 min later (Fig. 3), therefore values quoted for the heel–MG response were taken 13 min after mustard oil was applied and those for toes–flexor reflexes were taken 15 min afterwards. In the control (no drug) group, the first conditioning stimulus decreased responses in the heel–MG, toes–TA and toes–ST reflex pathways to a median of 77% (IQR 43–82%), 70% (IQR 46–76%) and 73% (IQR 43–78%) of pre‐mustard oil levels, respectively, and these decreases lasted for 47 min (IQR 28–61 min), at least 63 min (IQR 63–63 min) and at least 63 min (IQR 27–63 min), respectively. The corresponding values in the group to be given RX 821002 were 65% (IQR 52–80%), 43% (IQR 33–53%) and 60% (IQR 52–80%) of controls for at least 61 min (IQR 34–61 min), at least 63 min (IQR 63–63 min) and at least 63 min (IQR 7–63 min; Fig. 3). For all three reflex responses, these effects of mustard oil were not significantly different between the two treatment groups (Mann–Whitney tests, *P *> 0.1).

Following administration of RX 821002 (100–300 μg, intrathecally), a mustard oil injection into the contralateral MG had no significant (Friedman's anova,* F* = 9.413; *P *> 0.3) effect on the heel–MG reflex, which tended to increase above pre‐injection levels rather than being subject to inhibition (Fig. 3). Similarly, mustard oil failed to induce any significant [Friedman's anova,* F* = 12.43 (TA), 5.813 (ST); *P *> 0.1 in each case] inhibition of either flexor reflex in the presence of RX 821002. However, when the contralateral MG muscle received a repeat mustard oil injection in the control (no drug) group, heel–MG responses were significantly (Friedman's anova,* F* = 24.15; *P *< 0.01) inhibited a second time to a median of 79% (IQR 32–98%) of pre‐mustard levels, as were TA and ST responses to toe stimulation [Friedman's anova,* F* = 29.63 (TA), 19.02 (ST); *P *< 0.05 in each case], which declined to a median of 56% (IQR 45–77%) and 59% (IQR 45–85%) of controls. The median duration of these decreases was for at least 61 min (IQR 45–61 min), at least 63 min (IQR 51–63 min) and at least 63 min (IQR 27–63 min) for the heel–MG, toes–TA and toes–ST responses, respectively. For all reflexes, these effects were not significantly different to those achieved with the first mustard oil stimulus (Wilcoxon tests, *P *> 0.3).

### The effect on reflex responses of mustard oil applied to the snout

**Figure 4:**
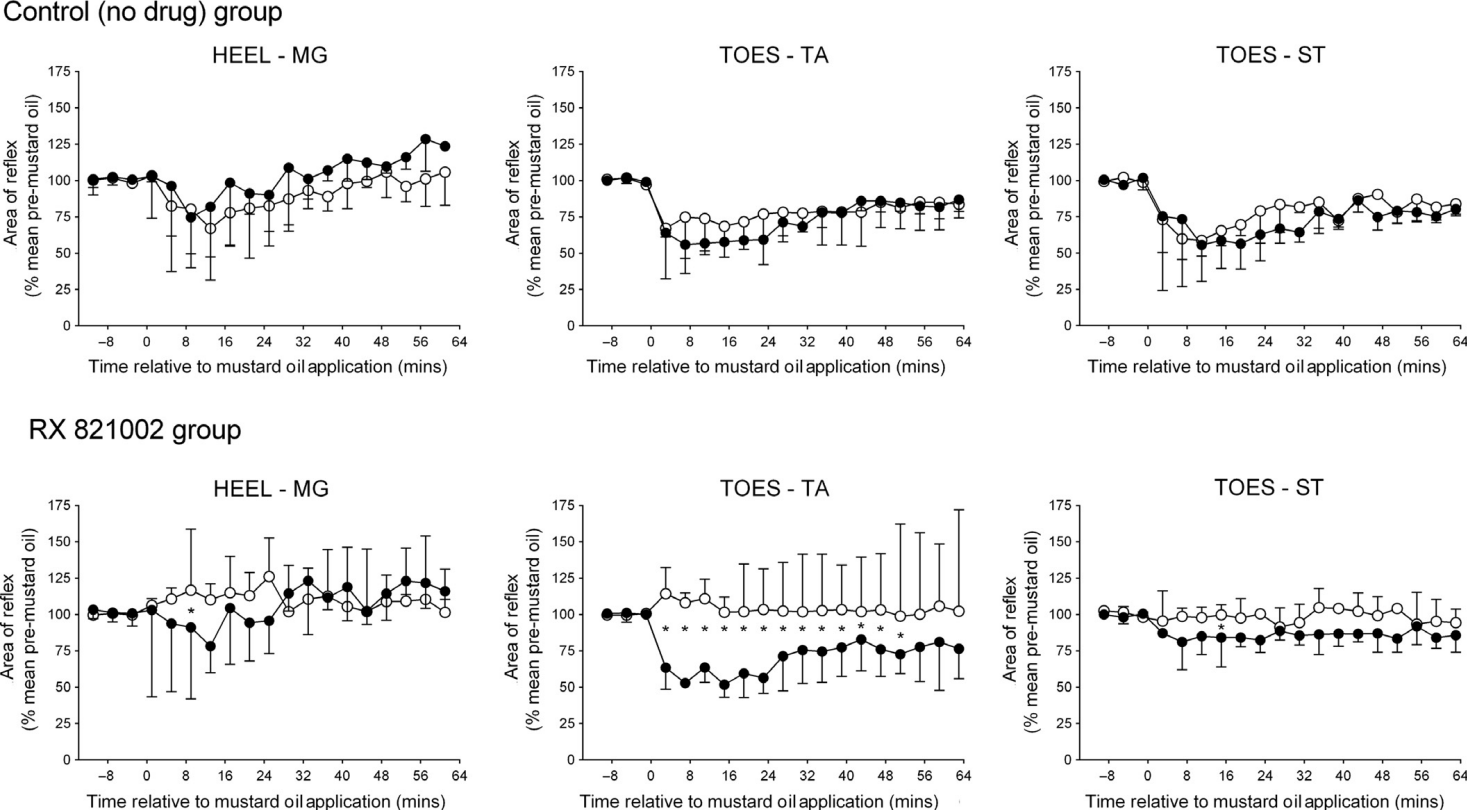
Effect of repeated mustard oil application to the snout (• first application; ∘ second application) on heel–medial gastrocnemius (MG), toes–tibialis anterior (TA) and toes–semitendinosus (ST) reflexes in the absence (top panels) or presence (bottom panels) of RX 821002 (100–300 μg, intrathecally) prior to the second injection. Each point on the graphs represents the median of nine (no drug group) or 10 (RX 821002 group) experiments, and the vertical lines are either 1st or 3rd quartiles. Mustard oil was applied at 0 min. *Indicates a significant difference in the effect of mustard oil at equivalent time points before and after intrathecal RX 821002 (Wilcoxon tests, *P *< 0.05).

Application of the first mustard oil stimulus to the snout resulted in a significant inhibition of all three reflexes responses [Friedman's anova, RX 821002 treatment group: *F* = 21.99 (MG), 29.30 (TA), 17.48 (ST); control (no drug) group: *F* = 17.54 (MG), 42.34 (TA), 27.08 (ST); *P *< 0.05 in all cases] that took 7–11 min to fully develop, so again quoted values for extensor and flexor reflex responses have been taken 13 min and 15 min after mustard oil, respectively. For the heel–MG reflex, a conditioning stimulus to the snout caused a decrease in responses to a median of 82% (IQR 31–102%) and 78% (IQR 60–123%) of pre‐mustard oil levels in control (no drug) animals and in the group to be given RX 821002, respectively (Fig. 4). The median duration of this inhibition was 17 min (IQR 0–33 min) for the control (no drug) group and 17 min (IQR 3–39 min) for the RX 821002 treatment group. The extent and duration of these inhibitions were not significantly different between the two groups (Mann–Whitney tests, *P *> 0.6). For the flexor reflexes, mustard oil to the snout inhibited the toes–TA and toes–ST responses to a median of 58% (IQR 56–66%) and 59% (IQR 39–79%) of pre‐mustard levels in the control (no drug) group, and this depression lasted for a median duration of at least 63 min (IQR 43–63 min) and 39 min (35–63 min), respectively. The corresponding values for animals that were going to receive RX 821002 were inhibition to 52% (IQR 43–72%) and 84% (IQR 64–86%) of controls for a duration of 63 min (IQR 35–63 min) and at least 35 min (IQR 23–63 min). Again, these values did not differ between the two treatment groups (Mann–Whitney tests, *P *> 0.2).

The inhibitory effect on the heel–MG reflex of mustard oil applied to the snout was completely abolished by pre‐administration of RX 821002, and in fact a small but significant (Friedman's anova,* F* = 19.56; *P *< 0.05) increase in the MG response was seen to a median of 117% (IQR 106–159%) of pre‐mustard controls (Fig. 4). This increase lasted for a median of 29 min (IQR 0–41 min). The presence of RX 821002 also led to mustard oil having no significant effect [Friedman's anova,* F* = 5.067 (TA), 5.719 (ST); *P *> 0.6 for both] on the response of TA or ST to toe stimulation (Fig. 4). However, in control (no drug) animals, application of mustard oil to the snout for a second time caused a significant [Friedman's anova,* F* = 33.30 (TA), 19.47 (ST); *P *< 0.05 for both] decrease in the toes–TA and toes–ST responses to a median of 68% (IQR 47–81%) and 65% (IQR 55–83%) of pre‐mustard levels for at least 63 min (IQR 63–63 min) and 43 min (IQR 31–59 min), respectively. These changes were not statistically distinguishable from those achieved with the first mustard oil stimulus (Wilcoxon tests, *P *> 0.4).

### The effect on reflex responses of mustard oil applied to the plantar MT joints

**Figure 5:**
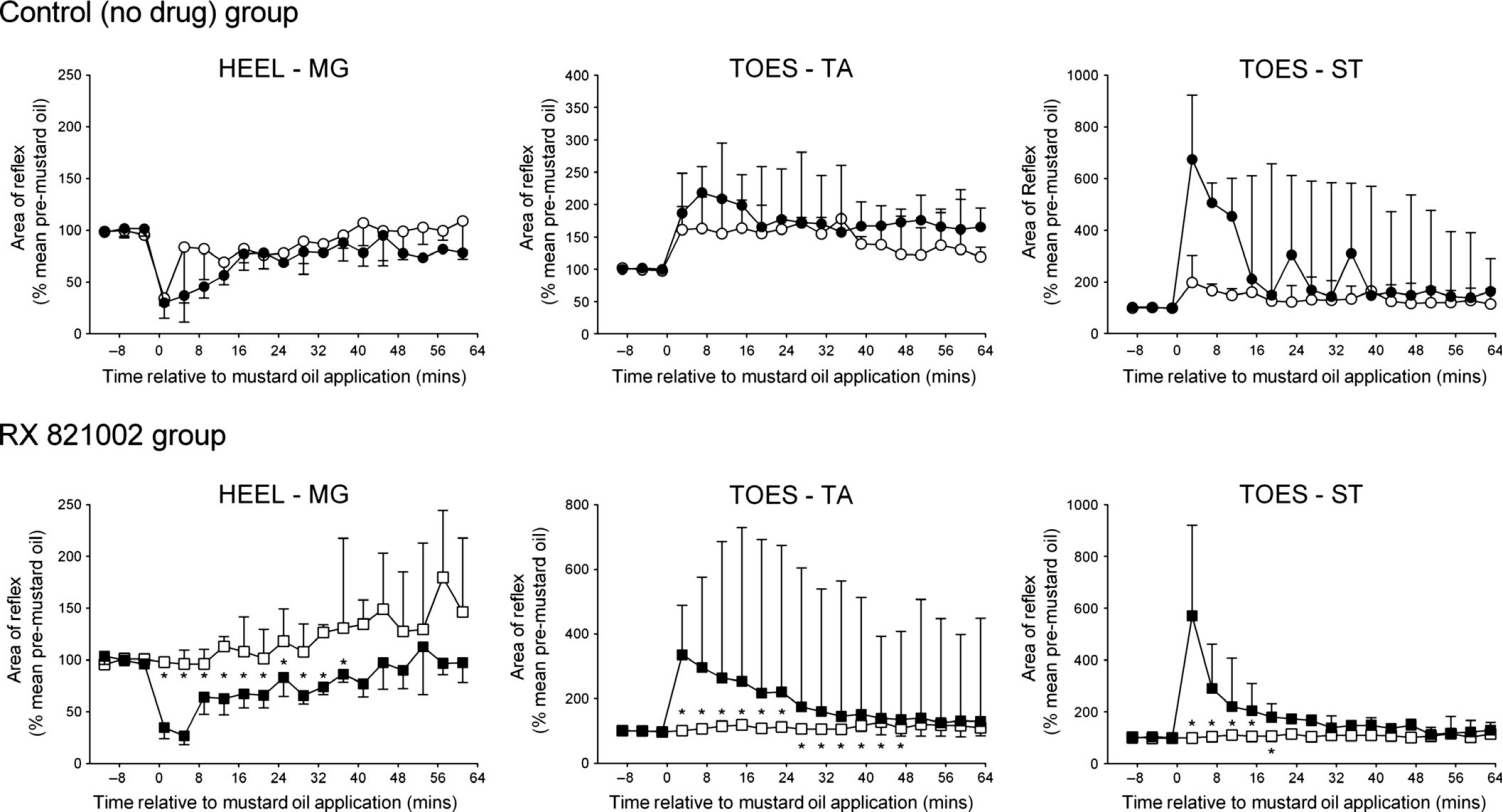
Effect of repeated mustard oil application to the plantar metatarsophalangeal (MT) joints (• first application; ∘ second application) on heel–medial gastrocnemius (MG), toes–tibialis anterior (TA) and toes–semitendinosus (ST) reflexes in the absence (top panels) or presence (bottom panels) of RX 821002 (100–300 μg, intrathecally) prior to the second injection. Each point on the graphs represents the median of nine (no drug group) or 10 (RX 821002 group) experiments, and the vertical lines are either 1st or 3rd quartiles. Mustard oil was applied at 0 min. *Indicates a significant difference in the effect of mustard oil at equivalent time points before and after intrathecal RX 821002 (Wilcoxon tests, *P *< 0.05).

In contrast to its application to off‐limb sites, the peak changes following mustard oil to the ipsilateral plantar MT joints occurred almost immediately post‐stimulus, therefore for this site values for the heel–MG reflex and the toes–flexor responses have been taken 1 min and 3 min after mustard oil treatment, respectively. In both control (no drug) and RX 821002 treatment groups, application of the first mustard oil stimulus to the MT joints caused the heel–MG reflex to significantly [Friedman's anova,* F* = 37.73 (RX 821002 treatment group), 37.03 (control (no drug) group); *P *< 0.0001 in each case] decrease to a median of 30% (IQR 15–49%) and 35% (IQR 24–47%) of pre‐mustard oil levels, respectively (Fig. 5). For control (no drug) animals, the median duration of this decrease was 33 min (IQR 21–53 min), whilst for those animals that were going to receive RX 821002 this time was 35 min (IQR 13–51 min). The size and duration of the mustard oil‐induced inhibition were not significantly different between groups (Mann–Whitney tests, *P *> 0.5). In contrast to the extensor response, the first mustard oil application to the MT joints significantly [Friedman's anova, RX 821002 treatment group: *F* = 39.31 (TA), 62.99 (ST); control (no drug) group: *F* = 37.03 (TA), 24.36 (ST); *P *< 0.005 in each case] facilitated the flexor reflexes for both treatment groups. In the control (no drug) group, increases were to a median of 187% (IQR 140–249%) and 675% (IQR 124–923%) of pre‐mustard oil levels for the toes–TA and toes–ST reflexes, respectively. In both cases, the median duration of these increases was at least 63 min (TA IQR 59–63 min; ST IQR 15–63 min). In the group to receive intrathecal RX 821002, the first mustard oil application caused the toes–TA response to be potentiated to a median of 336% (IQR 172–490%) of pre‐stimulus levels for at least 63 min (IQR 54–63 min), whilst the corresponding values for the toes–ST reflex were 571% (IQR 254–921%) of controls for a period of 57 min (IQR 51–63 min). The effects of this first mustard oil stimulus on each of the flexor reflexes did not differ between the two treatment groups (Mann–Whitney tests, *P *> 0.2).

When mustard oil was applied in the presence of RX 821002, an inhibitory effect on the heel–MG reflex was no longer observed, and instead a small but significant (Friedman's anova,* F* = 17.87; *P *< 0.005) increase in the MG response gradually occurred over time (Fig. 5). By contrast, a second mustard oil application to the MT joints in the control (no drug) group caused a significant (Friedman's anova,* F* = 18.01; *P *< 0.05) inhibition of heel–MG responses to a median of 34% (IQR 27–83%) of pre‐stimulus levels for 29 min (IQR 9–53 min), an effect that was not significantly different to the first mustard oil application to this site (Wilcoxon tests, *P *> 0.2). The presence of RX 821002 also abolished [Friedman's anova,* F* = 10.37 (TA), 6.320 (ST); *P *> 0.2 in both cases] the facilitatory effect of mustard oil on both of the flexor reflexes (Fig. 5).

However, when the second mustard oil stimulus was applied to the plantar MT joints in the control (no drug) treatment group, significant increases [Friedman's anova,* F* = 19.85 (TA), 28.44 (ST); *P *< 0.05 in both cases] in the toes–TA reflex, to a median of 161% (IQR 112–197%) of pre‐mustard oil levels, and the toes–ST response, to a median of 198% (IQR 108–303%) of pre‐mustard controls, were still observed. Both reflexes were enhanced for a median duration of at least 63 min (TA IQR 39–63 min; ST IQR 47–63 min). Comparison of the effects of the first and second mustard oil stimuli in this control (no drug) group indicated that they were not significantly different (Wilcoxon tests, *P *> 0.05).

### Effect of mustard oil on cardiovascular parameters

In the group of animals to be given RX 821002, application of the first mustard oil stimulus to the contralateral MG, the snout or the ipsilateral MT joints caused the mean arterial blood pressure to increase significantly (repeated‐measures anova,* F*_10,90_ = 9.231, *F*_10,80_ = 24.16 and *F*_10,90_ = 7.955, respectively, *P *< 0.0001 in each case), such that 5 min after the stimulus was applied, levels had risen by 4 ± 2, 26 ± 3 and 6 ± 3 mmHg, respectively. These increases lasted for an average of 19 ± 6, 37 ± 5 and 20 ± 7 min. The mustard oil stimulus also resulted in significant tachycardia [repeated‐measures anova,* F*_10,90_ = 17.38 (contralateral MG), *F*_10,80_ = 3.266 (snout) and *F*_10,90_ = 5.976 (ipsilateral MT joints), *P *< 0.005 for each site] and, 10 min after it was applied to the contralateral MG, the snout or the ipsilateral MT joints, mean heart rate had increased by 11 ± 2, 9 ± 4 and 4 ± 1 beats/min, respectively. The durations for which heart rate remained above control levels were 38 ± 8, 27 ± 9 and 23 ± 7 min. When mustard oil was applied to the same three sites in the control (no drug) group, statistically similar blood pressure and heart rate changes to the above were produced in each case (unpaired *t*‐tests, *P *> 0.1; Table 1). For both treatment groups, the pressor effect following mustard oil application to the snout was significantly greater than from the other two sites [one‐way anova followed by Tukey *post hoc* test, *F*_2,26_ = 25.50 (control (no drug) group), *F*_2,24_ = 19.15 (RX 821002 treatment group), *P *< 0.0001 for both, also see Harris & Clarke, 2003], but mustard oil‐induced tachycardia was statistically similar for all three sites [one‐way anova,* F*_2,26_ = 1.720 (control (no drug) group), *F*_2,24_ = 1.499 (RX 821002 treatment group), *P *> 0.1 in each case].

**Table 1:** Changes in arterial blood pressure and heart rate following mustard oil application to the contralateral MG, snout or ipsilateral MT joints

Site of application	No drug (control) group		RX 821002 treatment group	
	Pre‐mustard	Post‐mustard	Pre‐mustard	Post‐mustard
Contralateral MG				
1st application				
BP (mmHg)	84 ± 7	93 ± 6^a^	94 ± 4	97 ± 3^a^
HR (bpm)	269 ± 9	288 ± 9^a^	297 ± 9	308 ± 7^a^
2nd application				
BP (mmHg)	80 ± 4	87 ± 5^a^	86 ± 4	89 ± 5^a^
HR (bpm)	286 ± 8	294 ± 7^a^	305 ± 9	309 ± 8
Snout				
1st application				
BP (mmHg)	80 ± 5	109 ± 6^a^	87 ± 3	113 ± 3^a^
HR (bpm)	273 ± 7	285 ± 5^a^	299 ± 12	308 ± 9^a^
2nd application				
BP (mmHg)	74 ± 6	94 ± 6^a^	85 ± 6	99 ± 7^a^
HR (bpm)	279 ± 10	287 ± 10^a^	304 ± 9	312 ± 7^a^
MT joints				
1st application				
BP (mmHg)	81 ± 5	86 ± 6^a^	91 ± 3	97 ± 3^a^
HR (bpm)	280 ± 8	287 ± 8^a^	300 ± 8	304 ± 8^a^
2nd application				
BP (mmHg)	78 ± 5	81 ± 6^a^	82 ± 5	84 ± 6
HR (bpm)	285 ± 10	287 ± 11^a^	305 ± 8	305 ± 8

In the control (no drug) treatment group (*n* = 9), both mustard oil applications were in the absence of RX 821002, whilst the second mustard oil conditioning stimulus was performed after administration of RX 821002 (100–300 μg, intrathecally) in the RX 821002 treatment group (*n* = 10). Blood pressure and heart rate values are means ± SEM, and were taken 5 min and 10 min after mustard oil application, respectively.

BP, arterial blood pressure; HR, heart rate; MG, medial gastrocnemius; MT, metatarsophalangeal.

a A significant difference from the pre‐mustard oil state (repeated‐measures anova, see main text for statistical details).

**Figure 6:**
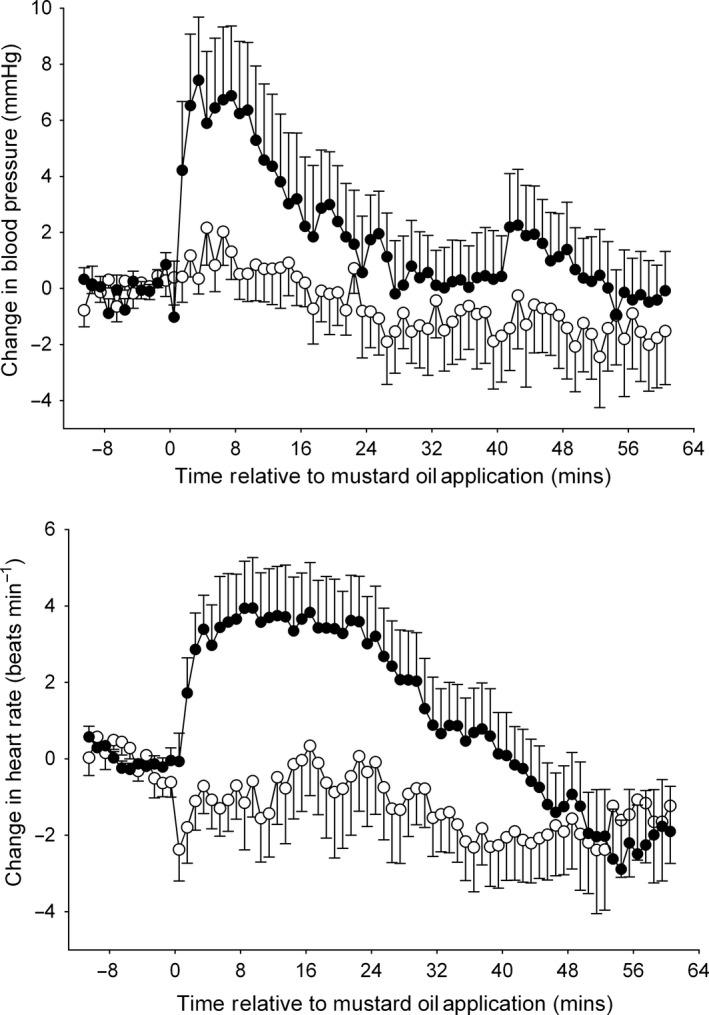
Effect of mustard oil applied to the metatarsophalangeal (MT) joints on mean arterial blood pressure (top panel) and heart rate (bottom panel) before (•) and after (∘) administration of RX 821002 (100–300 μg, intrathecally). Each point on the graph represents the mean of 10 experiments, and error bars are SEM. Mustard oil was applied at 0 min, and RX 821002 was administered 20–30 min before the second mustard oil stimulus was applied. Mustard oil caused a significant increase in blood pressure and heart rate before but not after administration of RX 821002.

Following intrathecal administration of RX 821002, mustard oil application to the snout still caused significant [repeated‐measures anova,* F*_10,80_ = 19.05 (BP), 3.032 (HR), *P *< 0.005 in both cases] increases in mean arterial blood pressure (by 14 ± 3 mmHg, 5 min after application) and heart rate (by 8 ± 5 beats/min, 10 min after application), which remained above control levels for 34 ± 8 and 20 ± 8 min, respectively. When injected into the contralateral MG, the second mustard oil stimulus again caused a significant pressor response (repeated‐measures anova,* F*_10,90_ = 7.018, *P *< 0.0001) such that 5 min after it was applied mean blood pressure had risen by 3 ± 1 mmHg and remained elevated above controls for 14 ± 6 min. This conditioning stimulus also caused the mean heart rate to increase by 4 ± 3 beats/min, although this rise was not statistically significant (repeated‐measures anova,* F*_10,90_ = 1.646, *P *> 0.1). However, mustard oil applied to the ipsilateral MT joints no longer caused a significant change in mean arterial blood pressure or heart rate [repeated‐measures anova,* F*_10,90_ = 0.8026 (BP), 0.8542 (HR), *P *> 0.5 for both; Fig. 6; Table 1]. In contrast to this, when the second mustard oil stimulus was applied to any of the three treatment sites in the control (no drug) group, blood pressure and heart rate significantly rose for a second time in all cases [repeated‐measures anova, contralateral MG: *F*_10,80_ = 6.062 (BP), 12.91 (HR); snout: *F*_10,80_ = 29.90 (BP), 10.48 (HR); ipsilateral MT joints: *F*_10,80_ = 3.286 (BP), 2.097 (HR), *P *< 0.05 in all cases].

## Discussion

This study has shown that in the pentobarbitone‐anaesthetized rabbit, prolonged inhibition of hind limb withdrawal reflexes following acute noxious stimulation of off‐limb sites is dependent on an intact α_2_‐adrenoceptor system in the spinal cord. In addition, α_2_‐adrenoceptors were found to be involved in both inhibition and facilitation of reflexes to noxious conditioning stimuli applied to the same limb. Taken together these data support the proposal that supraspinal noradrenergic pathways are involved in heterotopic inhibition as well as homotopic inhibition or excitation of spinal reflex excitability.

### Inhibition of hind limb reflexes by heterotopic noxious stimuli

In line with previous findings (Harris & Clarke, 2003), mustard oil stimulation of off‐limb sites caused inhibition of hind limb reflexes. Both flexors and extensors were inhibited even though reflexes to certain other muscles, for example plantar flexors of the digits, can be facilitated by such conditioning stimuli (Kalliomäki *et al*., 1992; Morgan *et al*., 1994; Morgan, 1999). This therefore has features in common with counter‐stimulation phenomena, such as DNIC (Le Bars *et al*., 1979a,b; Villanueva & Le Bars, 1995), which has been demonstrated on reflexes following noxious mechanical, thermal and electrical stimuli in rodents (Schouenborg & Dickenson, 1985; Kalliomäki *et al*., 1992; Falinower *et al*., 1994) and humans (Willer *et al*., 1984, 1989; Terkelsen *et al*., 2001; Ge *et al*., 2007), the effects lasting a number of minutes. In contrast, mustard oil, which selectively activates C‐fibre afferents (Woolf & Wall, 1986) via a specific agonistic effect at ankyrin‐type transient receptor potential (TRPA1) channels (Jordt *et al*., 2004), produced prolonged inhibition for up to at least 1 h (Harris & Clarke, 2003). As spatial extent and intensity of the conditioning stimulus are important factors influencing amplitude and duration of DNIC (Willer *et al*., 1984, 1989; Falinower *et al*., 1994), the area subjected to mustard oil was restricted to a few mm^2^ and, although not removed, evidence suggests it causes a nociceptive afferent barrage of only a few minutes duration (Cook *et al*., 1987; Harris & Clarke, 2003). Other chemical conditioning stimuli, for example capsaicin (Gjerstad *et al*., 2000), ethylchloride (Parsons & Goetzl, 1945) and formalin (Wen *et al*., 2010), similarly produce heterotopic inhibition of long duration, likely reflecting central changes. The benefit of widespread long‐lasting inhibition of normally protective reflex responses is unclear, but it may generate time for a co‐ordinated escape response when the animal has been injured and is under threat (Harris, 1996; LeDoux, 1996).

### Involvement of noradrenergic mechanisms in heterotopic inhibition

Blockade of spinal α_2_‐adrenoceptors by intrathecal RX 821002 prevented mustard oil‐induced heterotopic inhibition of reflexes. This result is supported by the previous finding that intravenous idazoxan attenuated inhibition of sural‐MG reflexes following electrical stimulation of high‐threshold afferents in either forelimb or the contralateral hind limb (Taylor *et al*., 1991). Possible peripheral effects of intrathecal RX 821002 (i.e. due to leakage into the circulation) are likely to be minimal, as it induces changes in reflexes and blood pressure in intact but not spinalized animals (Harris & Clarke, 1993; Ogilvie & Clarke, 1998), confirming a spinal site of action. Furthermore, systemic dexmedetomidine, an α_2_‐agonist, caused a reduction in DNIC/CPM inhibition (Sanada *et al*., 2009; Baba *et al*., 2012) via a proposed supraspinal site of action; an effect of RX 821002 supraspinally would therefore be predicted to enhance DNIC, which clearly was not the case in the present studies. Because mustard oil‐induced heterotopic inhibition of reflexes also cannot be generated in spinalized animals (Harris & Clarke, 2003), its effects are presumably due to noradrenaline released from descending supraspinal pathways which, by the simplest interpretation, acts at pre‐synaptic α_2_‐adrenoceptors on heel or toe primary afferents to reduce excitatory neurotransmitter release (Kuraishi *et al*., 1985; Takano *et al*., 1993; Ueda *et al*., 1995; Pan *et al*., 2002; Kawasaki *et al*., 2003) or postsynaptically to inhibit interneurons in the reflex arcs (North & Yoshimura, 1984; Sonohata *et al*., 2004; Lu & Perl, 2007; Gassner *et al*., 2009). Anatomical studies in many species, including the rabbit (Blessing *et al*., 1978, 1981, 1986), show descending fibres originate from the A5, A6 (nucleus locus coeruleus, LC and subcoeruleus, SC) and A7 noradrenergic nuclei in the brainstem (Dahlström & Fuxe, 1964), direct stimulation of which inhibits spinal neurons via α_2_‐adrenoceptors (Jones, 1991; Millan, 2002; Pertovaara, 2006). However, lesions incorporating these nuclei do not reduce DNIC, which instead appears to arise from subnucleus reticularis dorsalis in the caudal medulla (Bouhassira *et al*., 1990, 1992a,b, 1993, 1995). Some involvement of A5–A7 should not be entirely discounted though, as during acute hind paw inflammation, heterotopic inhibition involved LC/SC (Tsuruoka *et al*., 2004). Until now, investigation of spinal noradrenergic pathways in heterotopic inhibition of flexor vs. extensor reflexes has been minimal, but some evidence suggests noradrenergic mechanisms are involved in heterotopic inhibition of dorsal horn neurons (Gjerstad *et al*., 2000) and tail flick responses (Wen *et al*., 2010). However, similar to earlier studies (Taylor *et al*., 1991), these groups also reported the additional involvement of opioidergic pathways. The inhibitory pathways recruited may differ according to the conditioning stimulus (Wen *et al*., 2010) or the site from which DNIC is evoked (Taylor *et al*., 1991). Complete reversal of mustard oil‐induced effects by RX 821002 in the current studies suggests a dominance of noradrenergic influences in this anaesthetized preparation (see below). Further studies are required to identify the source of noradrenergic pathways involved.

### Involvement of noradrenergic mechanisms in homotopic conditioning stimuli

In contrast to broad inhibition of cutaneomuscular reflexes from off‐limb sites, consequences of conditioning stimulation of the same limb are more complex, being inhibitory or facilitatory depending on the area stimulated and reflex studied, as well as being influenced by descending pathways (Clarke & Harris, 2004). Hence, mustard oil to the MT joints caused significant inhibition of heel–MG reflexes but long‐lasting facilitation of toes–flexor responses (Clarke *et al*., 2001b; Harris & Clarke, 2003; Harris *et al*., 2004). These changes mirror modular organization of reflexes *per se* (Clarke *et al*., 1989; Schouenborg & Kalliomaki, 1990; Weng & Schouenborg, 1996; Andersen *et al*., 1999; Levinsson *et al*., 1999), and increase excitability of reflexes that protect the injured site whilst reducing those that aggravate the injury. The present studies have now found that both inhibition and facilitation can be completely abolished by intrathecal RX 821002, indicating involvement of noradrenergic pathways in differential homotopic inhibition or sensitization of certain spinal reflexes, hence in controlling appropriate reflex responses to injury at a particular site. As it is well established that α_2_‐adrenoceptors directly mediate pre‐ and postsynaptic inhibition not facilitation (Pertovaara, 2006), the mechanisms underlying this dual effect of RX 821002 are likely to be complex, with mustard oil‐induced facilitation involving a noradrenergic ‘enabling switch’, i.e. a disinhibitory process, allowing excitatory (sensitizing) pathways such as those involving glutamate and substance P to be expressed (Harris *et al*., 2004). Electrophysiological studies have shown α_2_‐adrenoceptors can mediate hyperpolarization (North & Yoshimura, 1984; Sonohata *et al*., 2004; Lu & Perl, 2007; Gassner *et al*., 2009) of putative γ‐aminobutyric acid (GABA)‐ergic inhibitory and glutamatergic excitatory interneurons, presenting a means by which bulbospinal noradrenergic pathways could suppress inhibition and facilitation of spinal transmission (and reflexes), respectively (Lu & Perl, 2007). Mustard oil‐induced up‐ or downregulation of activity in noradrenergic pathways could therefore strengthen or weaken these spinal effects, and evidence for such changes, from the LC/SC in particular (Tsuruoka & Willis, 1996; Tsuruoka *et al*., 2004; Viisanen & Pertovaara, 2007; Maeda *et al*., 2009), is seen in acute inflammation (Green *et al*., 1998; Omote *et al*., 1998; Mansikka *et al*., 2004) and nerve injury (Xu *et al*., 1999; Wei & Pertovaara, 2006; Rahman *et al*., 2008; De Felice *et al*., 2011; Hughes *et al*., 2013).

### Tonic effects and anaesthetic considerations

Tonic inhibition of spinal excitability may be mediated by supraspinal sites different to those involved in DNIC (Bouhassira *et al*., 1995). Certainly, spinal reflexes are subject to powerful tonic descending noradrenergic inhibition that can be alleviated by intrathecal α_2_‐adrenoceptor antagonists (including RX 821002), but not the α_1_‐antagonist prazosin, in decerebrated (Harris & Clarke, 1992, 1993; Clarke *et al*., 1998, 2000, 2001a) or alphaxalone/alphadolone (Saffan)‐anaesthetized rabbits (Ogilvie *et al*., 1999). However, in the present studies, when given by the same route and at similar doses, RX 821002 had no effect *per se* on any reflex. In addition, although RX 821002 caused a significant decrease in mean arterial blood pressure along with a significant increase in heart rate (likely a baroreceptor‐mediated compensatory response), previously pressor effects have been observed in decerebrated animals (Harris & Clarke, 1993; Clarke *et al*., 1998, 2000), presumably due to block of tonic descending noradrenergic inhibition of sympathetic preganglionic neurons (Coote & Lewis, 1995). In contrast, intrathecal RX 821002 had no effect on blood pressure in Saffan‐anaesthetized animals (Ogilvie *et al*., 1999). Taken together, these data indicate that mixed effects of α_2_‐adrenoceptor blockade on control of sympathetic and somatic motor outflows are observed depending on the presence and type of anaesthesia; in particular, that tonic noradrenergic descending inhibition is suppressed in pentobarbitone‐anaesthetized animals. Although DNIC is dependent on choice (Alarcón & Cervero, 1989) and depth (Tomlinson *et al*., 1983; Jinks *et al*., 2003) of anaesthesia, in the present experiments it is clear from the control (no drug) group that the anaesthetic regime was highly stable throughout and did not affect repeatability of heterotopic or homotopic conditioning stimuli. Therefore, whilst noradrenergic pathways may be particularly prominent in heterotopic inhibition in pentobarbitone‐anaesthetized rabbits, it does not diminish the fact these pathways can potentially be activated by noxious stimuli in awake behaving animals. This supports the previous observation that descending control of reflex excitability is a dynamic process (Harris & Clarke, 2003), and that anaesthetic contribution to the balance of supraspinal controls always needs to be considered.

### Cardiovascular responses to mustard oil

Mustard oil application to any site caused significant increases in mean arterial blood pressure and heart rate (likely due to activation of brain stem defence areas, for example the dorsolateral periaqueductal grey matter; Blessing, 1997), with the snout stimulus being particularly effective. However, reflex modulation was clearly not driven by these changes, as the greater cardiovascular effects of the snout stimulus were not reflected in a greater level of heterotopic inhibition of reflexes, and mustard oil to the MT joints clearly had a differential effect on reflexes. In addition, this latter site was the only one where mustard oil‐induced cardiovascular responses were blocked by intrathecal RX 821002 even though antagonism of α_2_‐adrenoceptors prevented its modulation of all reflex responses. This provides further evidence for differential activation of brain circuits by noxious stimuli applied to different parts of the body.

### Possible 5‐HT involvement in observed effects

RX 821002 is the 2‐methoxy derivative of idazoxan, which has no appreciable affinity for non‐adrenergic imidazoline receptors and high selectivity for α_2_‐ vs. α_1_‐adrenoceptors (Hudson *et al*., 1992; O'Rourke *et al*., 1994; Clarke & Harris, 2002). It is therefore possible to be confident that most, if not all, of the effects observed were via noradrenaline acting at α_2_‐adrenoceptors. However, it is possible some of the effect of RX 821002 could be due to its weak antagonistic actions at 5‐HT_1A_ receptors (Newman‐Tancredi *et al*., 1998; Ogilvie & Clarke, 1998; Clarke & Harris, 2002). Descending 5‐HT‐ergic pathways exist in the rabbit (Felten & Cummings, 1979; Howe *et al*., 1983; Yamada & Sano, 1985), and tonic inhibition of reflexes via 5‐HT_1A_ receptors has been shown (Clarke *et al*., 1996; Ogilvie *et al*., 1999). Studies in the rat suggest involvement of descending 5‐HT‐ergic pathways in DNIC (Dickenson *et al*., 1981; Chitour *et al*., 1982; Kraus *et al*., 1982) and modulation of spinal cord excitability (Millan, 2002). Future studies using intrathecal administration of selective 5‐HT receptor antagonists (e.g. WAY‐100635) could therefore investigate whether 5‐HT_1A_, or other 5‐HT receptor subtypes, are involved in heterotopic and homotopic modulation in this model.
